# Artificial Intelligence in Forensic Medicine and Toxicology: The Future of Forensic Medicine

**DOI:** 10.7759/cureus.28376

**Published:** 2022-08-25

**Authors:** Toshal D Wankhade, Sundeep W Ingale, Prakash M Mohite, Nandkishor J Bankar

**Affiliations:** 1 Forensic Medicine, Datta Meghe Medical College, Datta Meghe Institute of Medical Sciences, Nagpur, IND; 2 Microbiology, Jawaharlal Nehru Medical College, Datta Meghe Institute of Medical Sciences, Wardha, IND

**Keywords:** medicolegal autopsy, toxicology, crime investigations, forensic medicine, artificial intelligence

## Abstract

The predominance of artificial intelligence (AI) will be the next Industrial Revolution. All the fields of industry will be reshaped with AI. Forensic medicine and toxicology is an important branch for investigations of crimes and this branch has tremendous scope of development with the help of AI. Any science or process cannot stay away long from newer techniques. The traditional way of doing an autopsy and framing an opinion has a lot of limitations and these limitations can be overcome with AI. Various procedures of forensic medicine like analysis of toxins, collection of the various samples of medicolegal importance from body cavity, detection of pathological changes in various organs of the body, detection of various stains on the body, detection of a weapon used in crime, time since death calculations, etc. are the areas where AI will play a key role in framing the various opinions of medicolegal importance. AI can also be integrated into existing testing and analysis processes to make the whole procedure rapid and more accurate. In the future, AI might become a key part of forensic medicine and toxicology practice.

## Introduction and background

Today, the fourth Industrial Revolution is completely reshaping numerous industries. Artificial Intelligence (AI) has become a new cornerstone for all digital transformation initiatives [1]. In the past few years, we have witnessed a rise in the adoption of AI in multiple fields, including healthcare. AI is now being used to detect skin cancer, detect and monitor vital signs, and even assist doctors in making more accurate diagnoses [2]. There is a huge scope in the field of toxicology for AI to be used in the analysis of biological samples [3]. As AI can support forensic medicine experts and toxicologists with complex analytical tasks, it might also change the practice of forensic medicine and toxicology. In this review article, we explore how AI could impact the field of forensic medicine and toxicology, as well as what needs to happen for AI to have a profound effect on this space.

## Review

What are forensic medicine and toxicology?

Forensic medicine is the application of medical knowledge to law enforcement, criminal investigation, and the legal system. Toxicology is the study of the adverse effects of chemicals on human health. The main focus of toxicology is the study of the effects of both naturally occurring and man-made chemicals on the human body [4].

There are various sub-specialities of toxicology, like clinical toxicology, which deals with the human diseases caused by the effect of poisons on the human body. Medical toxicology deals with the treatment of poisons. Occupational toxicology deals with occupational hazards of various toxic materials on the human body. Analytical toxicology deals with the analysis and detection of poison. Toxinology is a branch of toxicology that deals with various toxins produced by living organisms that are harmful to the human body. Forensic toxicology applies this multidimensional knowledge of toxicology for the purposes of law and administration of justice [5].

The main focus of toxicology is the study of the effects of both naturally occurring and man-made chemicals on the human body. It encompasses a wide range of substances, including prescription medications, over-the-counter medications, alcohol, narcotics, illicit drugs, environmental toxins, etc.

Forensic medicine and toxicology are the foundations of forensic science. They are applied in a wide variety of scenarios and are commonly used in crime scene investigations and court cases. Forensic experts use a wide range of techniques for the purpose of identification and to determine if someone was poisoned, infected, had a certain medical condition, or died by violence. Techniques include gathering and analyzing DNA and fingerprints, examining blood and other bodily fluids, and examining hair, fibres, weapons, bones, and soil samples from a crime scene.

In this article, we have discussed various key points in the field of forensic medicine, where the application of AI will be of great help to forensic medicine experts.

AI in forensic medicine and toxicology

AI includes the building of smart machines that can perform tasks like human intelligence. The traditional way of doing an autopsy has many limitations, i.e., it requires experienced manpower in each and every case, some minute observations can be missed by a naked eye examination, and at some points person to person variation may occur while framing the opinions. The application of advanced technology in the field of forensic medicine is the need of the hour to perform a medicolegal autopsy in an accurate manner.

While performing the medicolegal autopsy, a forensic expert has to look for various points as per the need of the case to frame a proper opinion regarding the cause of death and to answer the queries of the investigating agency. These include proving the identity of an individual, externally examining various stain marks on cloths or body, identifying and collecting samples of body fluid, examination of wounds, etc. In an internal examination of the body, the forensic expert has to find out various pathological conditions of the organs to detect the cause of death; they have to look for various minor fractures and injuries, which are usually missed by naked-eye examination, but these injuries may have a role in causing the death of the person. They also have to look for any affected or inflamed area present in the body due to the effect of poison. They also have to examine various pieces of trace evidence like blood samples, seminal stains, and fingerprint marks. In all those areas of forensic medicine, AI can play a key role in aiding forensic experts to form more accurate, quick, and uniform opinions related to forensic case examination by comparing the data from their findings with the data available from machines. Similarly, weapon examination and calculation of time since death also are areas where AI can be helpful [6].

While reviewing the topic ‘AI in Forensic Medicine and Toxicology,’ we have gone through various pieces of literature and took references from various articles published on this matter in the standard journals. We identified some potential areas where AI can be applied by forensic experts in the field of forensic medicine, as described below. 

Identification is a very important aspect in the field of forensic medicine as it is required in the identification of criminals, identification of the unknown dead body, identification in mass disasters situations like earthquakes, bomb blasts, floods, etc. The traditional way of Identification involves anthropology, facial descriptions, tattoos, scars, body marks, etc. Fingerprinting and DNA analysis are newer techniques introduced nowadays. But with the advent of AI, machines can be used to establish identity. When a machine is provided with the input of various body parameters like facial features, retinal patterns, and fingerprints, the machine will store this data electronically. By using these parameters of individuals, which are already stored electronically in the machine, the machine can establish the identity of individuals by using AI [7,8]. Fingerprint data is already being used widely for this purpose for marking the attendance of students at education institutes and the attendance of employees at corporate offices.

A machine can be fed with various parameters for establishing the identity; these include fingerprint pattern, iris pattern, facial features, gait pattern, DNA pattern, palm print, and voice pattern. Identification of the individual by this biological data is known as biometry. When the particular pattern of biometry of the individual, e.g, a fingerprint, is brought in front of the machine, it will quickly recognize that individual by matching the biometric pattern which is already stored in the machine. Thus, individual identity can be established by matching the biometric pattern put in front of a machine with the biometric data of the individual already stored at the machine. To apply this technique to a large population, the machine will require biometric data of all individuals of a particular locality. This biometric technique will be of great help in establishing the identity of an individual. However, there will be many infrastructure- and resource-related challenges while providing the biometric data of a vast population to the machine. [9]

Stains on the body or various colour changes on the body are examined externally by the naked eye. AI can have a key role here. For example, stains of seminal fluid may be present in cases of sexual assault. Sperm detection is an important factor in such cases for criminal investigation and framing the charge. A naked-eye examination can miss this important stain mark, or many times, collected samples are inadequate to detect the sperm in the sample. AI microscopy imaging technique can visualize sperm stains more accurately by using the deep convolution neural networks technique. This automated image analysis technique identifies objects and substances in images; this technique can also be used in the detection of fingerprints on a surface or blood spatter patterns on clothing [10].

Colour changes in bruises are important for the calculation of the age of the injury. Colour interpretation by the naked eye has limitations. Computer-aided colour detection techniques can analyze the colour more accurately from the wound. The machine is provided with various graphs related to trauma time intervals and changes in wound colour pattern. By comparing the available data, the machine can interpret the time interval of injury in a very accurate manner [11]. 

AI-enhanced virtual autopsy is a newer trend being used in the field of forensic medicine while performing the autopsy. Machine learning tools will take the images of the body with the help of a CT scan or MRI technique. The machine will itself identify the pathological condition of an organ from the images by comparing it with the vast amount of input data provided to the machine [6]. The machine will process the data and will draw the conclusion regarding the disease condition of the organs, and the cause of death can be framed. Opinions regarding organ pathology, minor fractures, deep injury, and inflammation of the tissue can be framed. The technique can also give an opinion regarding the kind of weapon used by analyzing the dimensions of injury and comparing it with the various pattern of injury by different types of weapons. The technique will also help in the collection of samples from the specific pathological sites of organs and it will help in establishing an accurate diagnosis of disease. 

Time since death estimation is an important factor in the field of forensic medicine and criminal investigation. There are various markers in blood which can be used to predict the time since death. These blood markers can be processed through an AI device and thus accurate opinion regarding the time since death can be obtained. Biomarkers which can be used in this technique are lactate dehydrogenase (LDH), pH of blood, triglyceride, cholesterol, sodium, potassium, etc. After death, decomposition of the body starts and the levels of biomarkers change with proportion to time duration. With the help of available statistical data, changes in the levels of biomarkers can be interpreted in the form of time since death by an AI device [12,13].

There are various modern methods for chemical analysis available for processing various body samples for forensic toxicological analysis. Some of the techniques used are photo spectrometer, chromatography, neutron activation analysis, high-performance liquid chromatography, etc. Though the techniques are advanced, human error can result in a wrong analysis of the sample; these analytical methods are complicated and expert staff is required for proper analysis. To avoid this, AI can play a key role. A set of algorithms is provided to a machine on the basis of which the machine can analyze the sample more accurately and the method will be less time-consuming compared to the conventional way of analysis [14]. AI can also be combined with robotics to automate some aspects of toxicology testing. For example, robots can be used to accurately collect and transport samples. AI promises a range of benefits for forensic experts, from increasing efficiency and reducing costs to promoting accuracy and enabling new types of toxicological analysis. 

Apart from this, AI has also potential uses in the following areas: (i) Disease Diagnosis, where AI can help with the diagnosis of diseases using medical images, such as CT, MRI, and PET scans [15], (ii) Disease Surveillance, where AI can be used to track unusual disease outbreaks. This includes monitoring the spread and evolution of pathogens, such as bacteria, viruses, fungi, and other infectious diseases [16], (iii) Forensic Autopsy and Pathology, where AI can help determine pathological causes of death. Autopsy and pathology reports are often long, tedious documents. AI can help shorten these documents by flagging key findings and conclusions [6], (iv) Toxicology Analysis and Forensics, where AI can be used to investigate the presence of drugs and poisons in biological samples, such as blood, urine, hair, saliva, and skin, (v) Drug Abuse, where using drug testing software, AI can identify trends in the drug abuse landscape. AI can also be used to flag abnormal and unusual usage patterns [17].

Omics data mining using AI

Omics is the suffix used in various branches of biology such as genomics, proteomics, metabolomics, toxicogenomics, etc. Omics technologies involve a large amount of scientific data which can be utilized in the forensic field for calculation of postmortem interval, diagnosis of disease, and analyzing drug abuse and poisoning cases. Studies in every forensic sector are increasingly using omics data from different platforms for research purposes as well. [18]. For example genomics study can be utilized for estimation of wound age by studying DNA microarray analysis of the skeletal muscle specimen in case of injury [19]. The advancement of biological and medical research has been aided by the advent of omics technologies, which produce large amounts of data in the areas of gene expression, protein measurement, metabolite levels, and microbial interaction. Omics data can be integrated with machine learning tools, and later on, this machine data can be applied in the field of forensic medicine for detecting various biomarkers and diagnosis of disease [20]. Studies on the subject of forensic medicine can utilize various kinds of data, information analysis, and mathematical modelling based on omics technologies. For example, the time-dependent expression of mRNA in contused muscle will help in the establishment of the age of contusion injury based on genomics data. [21].

Future forensic research will focus on multi-omics combined analysis and AI algorithm modelling. The process and regulatory mechanism of complex diseases can be more thoroughly and methodically reflected by the joint analysis of numerous omics using AI-enabled machines. [22].

Limitations of AI in forensic medicine and toxicology

Training of Machine 

An AI machine requires a large amount of data feed for accurate interpretation. Data should be in large quantities and of high quality for the machine to be able to learn from the data and for it to be trained in various key areas of forensic medicine by forensic experts so that it can interpret the finding in post-mortem examination and give a sound opinion. Forensic medicine experts must first manually annotate images and documents with key findings, conclusions, and other details to machines. For this, initially, a lot of effort needs to be made by forensic experts. 

*Ethics and Regulations* 

AI is a powerful technology that can promote efficiency and accuracy. However, at the same time, its implementation raises ethical questions and challenges existing regulatory environments. Some people argue that using AI will impede the traditional practice of human judgment.

*Trust* 

Forensic medicine experts need to build trust with their clients and stakeholders. They need to show the public that their work is accurate and trustworthy. Whether the opinion given by the machine will be trusted by the judiciary, investigating agency, and the common public will be a matter of great concern. Forensic experts need to explain to their clients, i.e., the judiciary and the investigation agency that the opinion derived from AI is accurate and trustworthy.

*Infrastructure* 

Forensic medicine experts need to have the right equipment and infrastructure to implement AI. This includes high-performance computing infrastructure and large data storage systems. All the processes will be very costly. Whether poorer nations will be able to afford such setups is also a matter of great concern. 

*Human Interaction* 

AI is simply an automated tool. It cannot replace human interaction and expertise. Every data fed to the machine will require human efforts. So, initially, for training the machine a lot of effort will have to be expended by forensic experts to train the machine and also regular updating of the data is a must. 

*Interoperability* 

Different AI tools might not be able to interact with one another. This might lead to a situation where silos of data are created. It will lead to duplication of efforts.

Medicolegal Implication

In a court of law, every documentary evidence needs to be testified by the expert orally. Hence, whether the AI-derived opinion will be accepted as evidence in a court of law is the most important matter of concern in the field of forensic medicine.

The Scenario in Developing Countries

In a developing country like India, it is a known fact that advanced healthcare facility is out of reach of a large section of the population. Hence, developing high-tech infrastructure in the field of forensic medicine will be very difficult for policymakers. 

## Conclusions

AI promises a range of benefits for forensic medicine experts, in the field of autopsy and toxicological analysis. The main challenge to implementing AI in forensic medicine and toxicology is finding high-quality data for training the machine. So initially a lot of effort will be required from forensic medicine experts across the globe to provide highly accurate data to machines. The required data for the machines may include various autopsy findings with good supportive images and accurate opinions regarding the pattern of the injury; it will include various statistical inputs regarding biomarkers and also the analytical algorithm. Providing data will be time-consuming and time to time updation of the machine data will be required. But if the experts from the forensic field overcome these initial hurdles, the AI tool will have a potential advantage in the field of forensic medicine to frame various opinions of medicolegal importance. From a traditional morphological perspective, computer technology and AI algorithms will improve forensic investigation methods with more accuracy and promptness. With integration into existing testing and analysis processes, AI might become a key part of forensic medicine and toxicology practice. 

Another matter of concern regarding AI is its legal value in a court of law. The court may not consider an opinion derived from AI as conclusive proof, but this opinion can act as corroborative evidence as any machine depends on the data it is being fed. Even the judiciary needs to understand the working pattern of the machine before giving the judgement. In such a scenario, judges may take expert review for the accuracy of the machine. However, this development will occur with the advent of time and evolution in the process of AI use. 

Setting up high-tech infrastructure in the field of forensic medicine will be a big challenge for policymakers in developing countries like India as the healthcare facility is in a developing stage and centred only in urban areas. The current priority for developing countries is to make medicare accessible to all the population of each and every part of the country. So, in a developing country like India, considering the current scenario of the healthcare system, AI-aided forensic medicine practice can be started as a pilot project at select centres. After assessing its efficacy and usefulness, such a practice can be expanded to larger areas in a phase-wise manner. 
